# miRNAs as biomarkers for early cancer detection and their application in the development of new diagnostic tools

**DOI:** 10.1186/s12938-021-00857-9

**Published:** 2021-02-16

**Authors:** Leonardo J. Galvão-Lima, Antonio H. F. Morais, Ricardo A. M. Valentim, Elio J. S. S. Barreto

**Affiliations:** 1 Advanced Nucleus of Technological Innovation (NAVI), Federal Institute of Rio Grande do Norte (IFRN), Avenue Senador Salgado Filho 1559, Natal, RN 59015-000 Brazil; 2 Laboratory of Technological Innovation in Health (LAIS), Hospital Universitário Onofre Lopes (HUOL), Federal University of Rio Grande do Norte (UFRN), Campus Lagoa Nova, Natal, RN Brazil; 3 Division of Oncology and Hematology, Hospital Universitário Onofre Lopes (HUOL), Federal University of Rio Grande do Norte (UFRN), Campus Lagoa Nova, Natal, RN Brazil

**Keywords:** MiRNA, Biomarkers, Liquid biopsies, Prostate cancer, Cervical cancer, Breast cancer, Early cancer diagnosis

## Abstract

Over the last decades, microRNAs (miRNAs) have emerged as important molecules associated with the regulation of gene expression in humans and other organisms, expanding the strategies available to diagnose and handle several diseases. This paper presents a systematic review of literature of miRNAs related to cancer development and explores the main techniques used to quantify these molecules and their limitations as screening strategy. The bibliographic research was conducted using the online databases, PubMed, Google Scholar, Web of Science, and Science Direct searching the terms “microRNA detection”, “miRNA detection”, “miRNA and prostate cancer”, “miRNA and cervical cancer”, “miRNA and cervix cancer”, “miRNA and breast cancer”, and “miRNA and early cancer diagnosis”. Along the systematic review over 26,000 published papers were reported, and 252 papers were returned after applying the inclusion and exclusion criteria, which were considered during this review. The aim of this study is to identify potential miRNAs related to cancer development that may be useful for early cancer diagnosis, notably in the breast, prostate, and cervical cancers. In addition, we suggest a preliminary top 20 miRNA panel according to their relevance during the respective cancer development. Considering the progressive number of new cancer cases every year worldwide, the development of new diagnostic tools is critical to refine the accuracy of screening tests, improving the life expectancy and allowing a better prognosis for the affected patients.

## Background

Over the last decades, microRNAs (miRNAs) have emerged as important molecules associated with regulation of gene expression in humans and other organisms, expanding the strategies available to diagnose and handle several diseases. Briefly, miRNAs are small non-coding RNAs (21–25 nucleotides) and derived from coding and non-coding transcription units in genic (intronic or exonic) and intergenic regions [1, 2].

These molecules were initially described in nematodes and implicated in the regulation of genic expression by post-transcriptional mechanisms targeting complementary mRNAs and affecting several biological processes, as cell signaling, differentiation, proliferation, and activation/inhibition of apoptotic mechanisms [2–4]. Currently, over 38 thousand miRNAs sequences from 271 species were described and cataloged on the miRBase (http://www.mirbase.org), which 1917 sequences are from *Homo sapiens* and may represent an important source of data to understand complex cellular mechanisms and establish a molecular diagnosis of several diseases [5, 6].

In this sense, considering the miRNAs proprieties and their role in the post-transcriptional regulation of genic expression, along the recent years we observed a successive accumulation of new evidence from differential expression of miRNAs in physiological and pathological conditions—including in infectious diseases and during the cancer development [7–14].

Classically, miRNAs can modulate the genic expression acting directly by intracellular mechanisms or after their release into microvesicles, allowing the modulation of gene expression between different tissues [15–17]. Both intra- and extracellular miRNAs can be detected on tissue samples and biological fluids (as serum, plasma, urine, saliva, sweat, and tears), but currently, this methodology is poorly explored in personalized medicine as diagnosis strategy or therapeutic tool [18–20]. Thereby, this review explores the role of miRNAs on the maintenance of tissue homeostasis, during cancer development, the major strategies adopted to detect and quantify these molecules, and their potential application as biomarkers for early cancer detection using a tissue or minimally invasive samples. Taken together, these findings may contribute to the development of new diagnostic tools for the quantification of miRNAs using clinical samples and allowing the application of personalized medicine strategies.

The current paper summarizes the latest findings related to the application of miRNAs as biomarkers for cancer detection, notably breast, prostate, and cervical cancers. These cancers, despite their high incidence worldwide, resulting in millions of new diagnoses and deaths every year worldwide [21, 22], present only few studies summarizing the recent findings in this area. Besides, we take an overview of current strategies used to quantify these molecules in tissue samples and liquid biopsies and point out the major challenges to applying these strategies as a screening method for early cancer detection.

## The biological basis of miRNAs and their use as biomarkers of cancer development

### Role of miRNAs during tissue homeostasis, extracellular signaling and their implication on the development of cancer

During physiological conditions, miRNAs play a key role in the control of tissue homeostasis and cell signaling, acting as a post-transcriptional mechanism of gene expression. The coordinate function of these molecules associated with other mechanisms avoid the development of aberrant cellular proliferation, regulates the cellular differentiation and allows the fine regulation of mRNAs in response to endocrine hormones and other stimuli (e.g., cytokines, chemokines, infectious or stress conditions) detected in the cellular microenvironment [23–26].

**Fig. 1:**
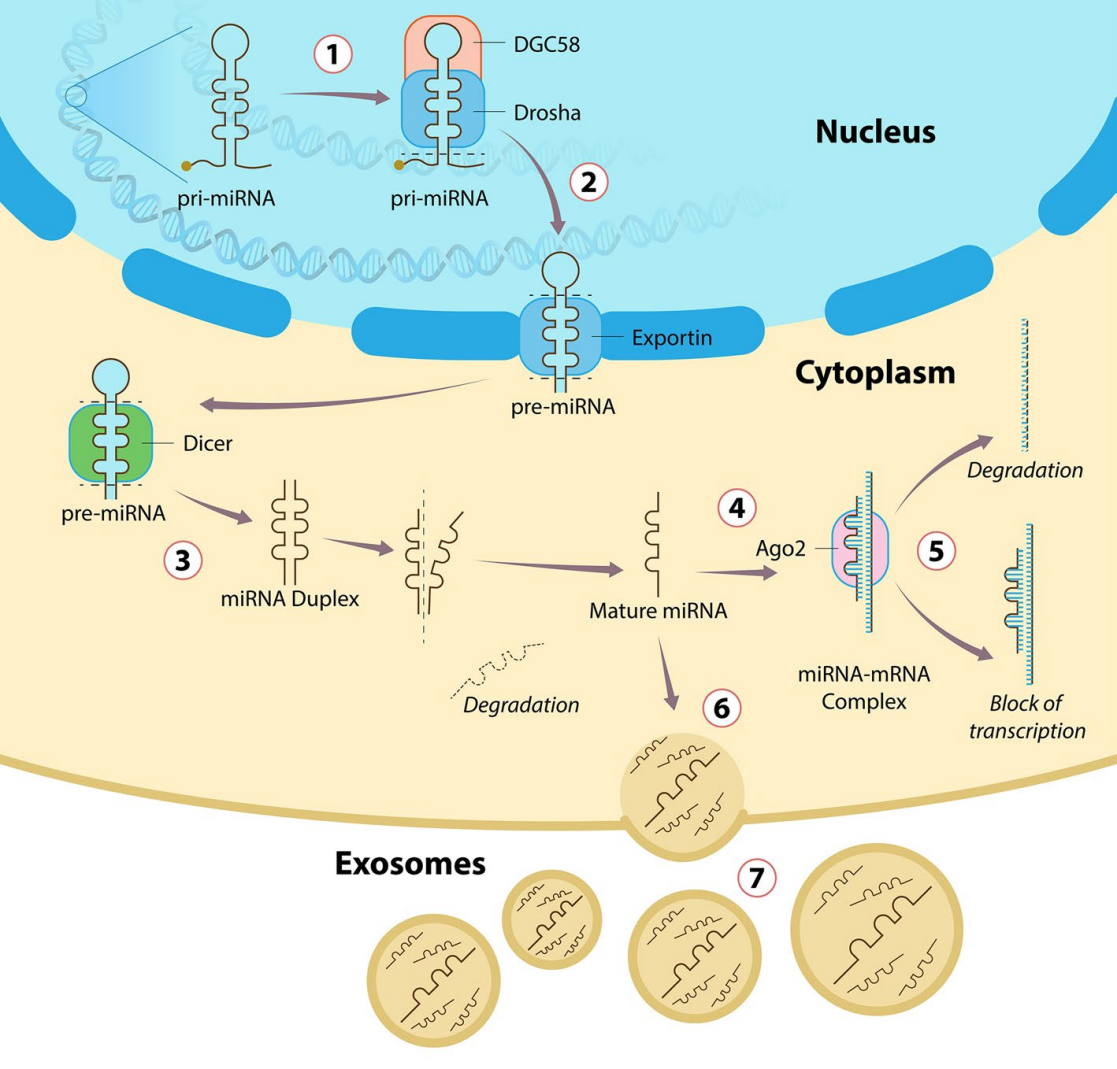
MicroRNA expression and function on post-transcriptional regulation of mRNA. (1) Mature miRNAs are initially expressed in the nucleus in the pri-miRNAs form, which is cleaved by DGC58/Drosha proteins, resulting in the pre-miRNA form. (2) The intermediary pre-miRNA form is exported to the cytoplasm. (3) The intermediary pre-miRNA form interacts with Dicer, responsible for the final cleavage of the hairpin loop and result in a 20–25 nucleotide dsRNA structure. (4) Mature miRNAs are released and form the miR-RISC protein complex. (5) miR-RISC protein complex interacts with the complementary mRNA strand and the acts on post-transcriptional regulation mechanisms. (6) Mature miRNAs are released into exosome vesicles. (7) Mature miRNAs in exosomes can be detected in several biological fluids (as plasma, saliva, or urine)

Typically, miRNAs are expressed in the precursor form (pri-miRNAs) by RNA Polymerase II in the nucleus and partially cleaved by DGC58/Drosha proteins, resulting in the pre-miRNA form, as represented in step 1 of Fig. 1. This intermediary (70–100 nucleotides) is exported to the cytoplasm by Exportin 5 proteins (step 2) and interacts with Dicer, which will be responsible for the final cleavage of the hairpin loop and results in a 20–25 nucleotide dsRNA structure (step 3). Finally, mature single strain miRNAs are released after the action of helicases and interact with Argonauts proteins (AGO1 and AGO2), resulting in the formation of the miR-RISC protein complex (step4), which stabilizes the following interaction with the complementary mRNA strand and acts on post-transcriptional control of gene expression, driven the mRNA for degradation or silencing (step 5) [27]. Additionally, mature miRNAs can be released into exosome vesicles (step 6) and affect the tissue microenvironment or present an endocrine action after being secreted on biological fluids (as plasma, saliva, or urine), resulting in local or systemic effects (step 7) [28–30].

Despite being systemically secreted by different cell types, the miRNAs expression follows a tissue-specific pattern, evidencing the key role of these molecules on cell differentiation and control of homeostasis [31]. Over recent years, this feature has been explored to map the miRNA expression profile in homeostatic conditions and how infections or other diseases can modulate these molecules. In this sense, Ying and colleagues evidenced that miRNAs (notably miR-155) released by adipose tissue macrophages in exosome vesicles play an important role in insulin sensitivity in vivo and in vitro and modulate the cellular glucose uptake [32]. Several miRNAs also have been implicated in the fine regulation of other endocrine signaling pathways by up- or downregulation of estrogen receptors ER-$\alpha$ and $\beta$ [33]. The expression of these molecules can also be influenced by the circadian cycle and contribute to the development of other non-cancer conditions, such as neurological, cardiovascular, and infectious diseases [7–14].

Although being progressively explored in basic research and their potential as a therapeutic tool, targeting specific genes and blocking the over/aberrant gene expression, miRNAs still is under adopted in clinical practices in personalized medicine—whether due to costs, safety or technical issues [34–36]. Currently, the NIH (clinicaltrials.gov) registered over 850 miRNA-related studies and their use for the development of new diagnostic tools or exploring their potential as a therapeutic approach to several diseases, such as sepsis, autism, diabetes mellitus 2, muscular dystrophy, amyotrophic lateral sclerosis, and cancer.

### How the changes in the miRNA expression allow the subversion of the tissue microenvironment and contribute to cancer development?

The dynamic balance between the apoptotic mechanisms and controlled cellular proliferation is critical to prevent the development of potentially malignant cells and keep the immune surveillance ready to act when an aberrant proliferation is detected. Usually, the host immune response activates cellular mechanisms mediated by NK and CD8+ T cells to eliminate unwanted cells and control the disordered cellular proliferation without damage the affected tissue. In this context, miRNAs are continuously expressed in rest or proliferating cells (including those related to the development of the immune response) and act to control the disordered cellular replication and differentiation, allowing the maintenance of tissue homeostasis or controlled response to stress or injuries [27]. However, when this tight control does not eliminate all detected threats by apoptotic and/or necrotic mechanisms or aberrant cells avoid the development of immune response, it can result in cancer development [37–39].

Several studies have implicated the changes in the miRNA expression pattern with the alteration observed in tissue microenvironment since the early stages of tumoral progression, allowing the formation of new blood vessels, the aberrant cellular proliferation and blocking the development of the immune response (e.g., inducing local Tregs cells and tumor-associated macrophages) [40–42]. Typically, these alterations occur without any manifestation of clinical symptoms or detection by traditional screening methods [19, 43]. In both contexts, several miRNAs are critical to block the function of cellular sensors and allow the uncontrolled proliferation or indicate the aberrant proliferation, recruiting immune cells to the affected tissue and avoid the tumoral development.

Among the main miRNAs related to the tumoral development, the overexpression of miR-21 was associated with the occurrence of several types of cancer (such as glioblastoma, breast, and gastrointestinal cancer) [44–46]. According to Xu et al. [47], this molecule has oncogenic property by targeting critical genes (as PTEN, RECK, and Bcl-2), allowing the aberrant cellular proliferation and tissue invasion of malignant cells. On the other hand, the downregulation of other miRNAs (as miR-141) is associated with cell proliferation and invasion in breast, colorectal, and prostate cancer [48–50], while several studies associate the regulation of p53 expression by the miRNAs [51–54]. Taken together, the dynamic balance mediated by several miRNAs between fine regulation of oncogenes and tumor suppressor genes act as a key regulator of tissue homeostasis and may indicate the early stages of tumoral development even when these alterations are not detected by traditional screening methods [55–57].

According to the recent estimates from Bray et al. [39], the number of new cancer cases is expected to affect up to 24.6 million people by 2030 and resulting in 13 million cancer-related deaths. In this sense, accurate early diagnosis is critical to the establishment of adequate therapeutic protocols, save lives, and improve the life expectancy of diagnosed patients [16, 58]. In recent years, the understanding of biochemistry and metabolic alterations induced during the aberrant cellular proliferation allowed the development of new chemotherapeutic agents, reducing the side effects and improving the therapeutic protocols [59–61]. However, since the description of the epigenetic mechanisms and the influence of miRNAs on post-transcriptional regulation events, a new era of molecular targets emerges as potential therapeutic agents and/or tissue-specific biomarkers of the development of tumoral cells [62–64].

This analysis leads us to the hypothesis that a complex miRNA panel may have an important role in the cancer diagnosis. However, currently there is no consensus on which molecules should be analyzed and how they can be applied as a biomarker of early cancer development, allowing the clinical staff to start the treatment in the initial stages of the disease and improving the quality of life from diagnosed patients.

### miRNAs as biomarkers of early cancer development: changes in the expression pattern detected in tissue samples and minimally invasive liquid biopsies

Although there is a lack of consensus on miRNA panel to detect the cancer development, this review summarizes the major findings related to the role of miRNAs during cancer development and the top 20 molecules related to breast, prostate, and cervical cancers. The current paper is not intended to present a definitive panel but to systematize our knowledge in the application of miRNAs as biomarkers of cancer development, and its importance in the rise of new strategies of diagnosis.

Considering all recent experimental and clinical data accumulated during research in physiological and pathological conditions, the quantification of miRNAs may be a powerful tool during the establishment of early cancer diagnosis, the evaluation of prognosis, and predictive biomarkers [58, 65]. Over recent years, changes in the pattern of miRNA expression serve as a molecular signature and have been used as a complementary tool to consolidate the cancer diagnosis [66–68]. This property allowed the identification of multiple primary and metastatic cancers by the origin of the tumoral tissue [69–73].

Besides the indication of cancer development with high sensibility and specificity, miRNAs also can be applied to confirm the initial pathological classification and to indicate the prognosis associated with cancer development [74, 75]. This approach may be used directly from tissue samples even during retrospective studies with formalin-fixed paraffin-embedded (FFPE) specimens archived for long periods or using minimally invasive methods [76].

However, despite these advances, currently, only a few studies associate the miRNA expression in tissue samples with the miRNAs released into exosomes, observed using minimally invasive liquid biopsies (as peripheral blood, saliva, urine, and tears) [15–17]. Although several basic studies evaluated the miRNAs present into vesicles, this gap hampers the use of miRNAs as a new strategic diagnostic tool, considering the needed for tissue samples and the use of specific bioinformatics tools to perform the clinical interpretation of results.

Currently, there is a lack of consensus on miRNA panels to detect the cancer development, but we must consider all published data to find which biomarkers may be useful to early detection of each type of cancer. In this sense, despite the suggestion present in the current paper, we encourage the development of new studies to adequate the targets miRNAs according to the genetic background of each population and the type and stage of cancer development.

Considering these findings, the development of a new tool to quantify miRNAs in clinical samples will be useful to detect several cancers and other diseases, including those that affect the CNS, once these molecules can cross the blood–brain barrier and indicate the occurrence of cellular alterations, in a complementary way to the traditional imaging exams [77–79]. Additionally, despite the current difficulties to correlate the serum miRNA profile to the tissue-specific tumoral alterations, the establishment of a well-defined panel and the adequate bioinformatics tools may be useful to distinguish between similar histological/phenotypical subtypes, providing an additional molecular classification using minimally invasive samples with high sensitivity and specificity [80–82].

### Preliminary panel of miRNA expression related to breast cancer development

As previously discussed, several miRNAs are identified as potential biomarkers of breast cancer development, as evidenced in Table 1. Among these, several act as onco-miRs (allowing the aberrant cellular proliferation). Some of these are represented for miR-21, miR-26a, miR-155, miR-221/miR-222, and miR-495, which are related to tumor proliferation and angiogenesis [46, 83–91].

**Table 1:** Preliminary panel for miRNA quantification related to breast cancer development

miRNA	Cellular function	References
let-7 family	Inhibition of cell proliferation, migration, and metastasis	[46, 83]
miR-1	Inhibition of tumor growth and metastasis	[97, 171]
miR-21	Promotion of cellular proliferation and tumor angiogenesis	[46, 83, 84]
miR-26a	Promotion of cell proliferation and differentiation in several tissues	[85, 86]
miR-100	Inhibition of tumorigenesis, cell proliferation and signaling	[98, 99]
miR-125b	Inhibition of cellular proliferation and induction of metastasis	[83, 100, 172]
miR-126	Inhibition of cell invasion by ADAM9 downregulation	[103, 104]
miR-145	Inhibition of cellular proliferation, migration, and tumor angiogenesis	[83, 105, 106]
miR-155	Promotion of cellular proliferation and tumor angiogenesis	[83, 87]
miR-195	Regulation of apoptosis and inhibition of tumor invasion	[111]
miR-199a	Inhibition of tumor invasion and metastasis	[84]
miR-200c	Regulation of apoptosis and metastasis	[29]
miR-203	Inhibition of tumorigenesis, cell proliferation, and signaling	[173]
miR-210	Response to hypoxia and stress conditions	[107, 174, 175]
miR-221/miR-222	Promotion of cellular migration and proliferation	[88–90]
miR-298	Response to hypoxia and control of cell proliferation	[108]
miR-331	Regulation cell proliferation, apoptosis, and inhibition of tumor invasion	[111]
miR-335	Regulation of BRCA1 expression, inhibition of cellular proliferation and migration	[109, 110]
miR-340	Regulation cell proliferation and inhibition of tumor invasion	[176]
miR-495	Promotion of cellular migration, proliferation, and response to hypoxia	[91]

On the other hand, several miRNAs (as those from let-7 family, miR-1, miR-100, miR-125b, miR-126, miR-145, miR-195, miR-199, miR-200c, miR-203, miR-210, miR-298, miR-331, miR-335, and miR-340) are implicated in the control of cell cycle, response to hypoxia and stress conditions, and induction of apoptotic mechanisms [46, 79, 92–110].

Additionally, McAnena et al. [111] observed that circulating miR-332 and miR-195 may be used to differentiate between local and metastatic breast cancer, while Sathipati et al. [112] suggest that 34 miRNAs can be used to classify the early and the advanced stage of breast cancer progression. These findings reinforce that a small number of miRNAs can be used as biomarkers to risk prediction or prognosis of breast cancer development [113–115].

### Preliminary panel of miRNA expression related to cervical cancer development

Similarly to breast cancer, several miRNAs are associated with the inhibition of cellular proliferation and migration (as miR-10b, miR-32, miR-124, miR-138, miR-143, miR-146a, miR-192, miR-214, miR-328, miR-429, miR-466) [64, 116–125]. Other studies associated the expression of miR-15b, miR-17, miR-21, miR-124, miR-130a, miR-218, miR-409, miR-432, and miR-454 with the regulation of cell differentiation and development of cervical cancer [118, 119, 126–130]. Table 2 summarizes the top 20 main miRNAs and their cellular function implicated in the development of cervical cancer.

**Table 2:** Preliminary panel for miRNA quantification related to cervical cancer development

miRNA	Cellular function	References
miR-10b	Inhibition of cellular proliferation and invasion	[116, 117]
miR-15b	Regulation of cellular proliferation and migration	[126]
miR-17	Promotion of cellular proliferation and tumor angiogenesis	[118]
miR-21	Promotion of cellular proliferation and tumor angiogenesis	[127]
miR-27b	Induction of cell proliferation, migration, and invasion	[177]
miR-32	Inhibition of cellular proliferation and migration	[118]
miR-124	Regulation of cellular differentiation and migration	[119]
miR-130a	Promotion of cellular proliferation and tumor angiogenesis	[128, 129]
miR-138	Inhibition of cellular proliferation and migration	[116]
miR-143	Regulation of apoptotic mechanisms	[120]
miR-146a	Regulation of inflammatory responses and cell differentiation	[121]
miR-192	Inhibition of cellular proliferation and migration	[64]
miR-214	Inhibition of cellular proliferation	[44]
miR-218	Regulation of cell differentiation and proliferation	[119]
miR-328	Inhibition of cellular proliferation	[123]
miR-409	Regulation of cellular proliferation and metastasis	[118]
miR-429	Inhibition of cell proliferation, migration, and invasion	[124]
miR-432	Regulation of cell proliferation and differentiation	[130]
miR-454	Regulation of cellular proliferation and metastasis	[118]
miR-466	Inhibition of cellular proliferation and control of apoptotic mechanisms	[125]

Several authors also evidenced the changes in the miRNA expression patterns in tissue samples and liquid biopsies, reinforcing the importance of studies using multiple types of samples. In this sense, Shukla et al. [118] observed that patients diagnosed with cervical cancer differentially expressed 119 miRNAs in tissue samples when compared to healthy controls, while 19 miRNAs were differentially expressed in serum and only 14 miRNAs were mutually altered in tissue and serum samples. A similar approach conducted by Nagy et al. [131] studying the miRNA expression in patients diagnosed with colorectal cancer identified that hsa-miR-3591-3p, hsa-miR-4506, hsa-miR-31, and hsa-miR-187 are similarly altered in tissue and plasma samples.

### Preliminary panel of miRNA expression related to prostate cancer development

Along the recent decade, several papers explored the miRNA expression from prostate cancer patients and compared these results with classical parameters (as PSA, biopsies results, and Gleason score), associating the tissue-restricted or circulating miRNAs expression with the occurrence of tumoral cells and the prognosis [132–134].

**Fig. 2:**
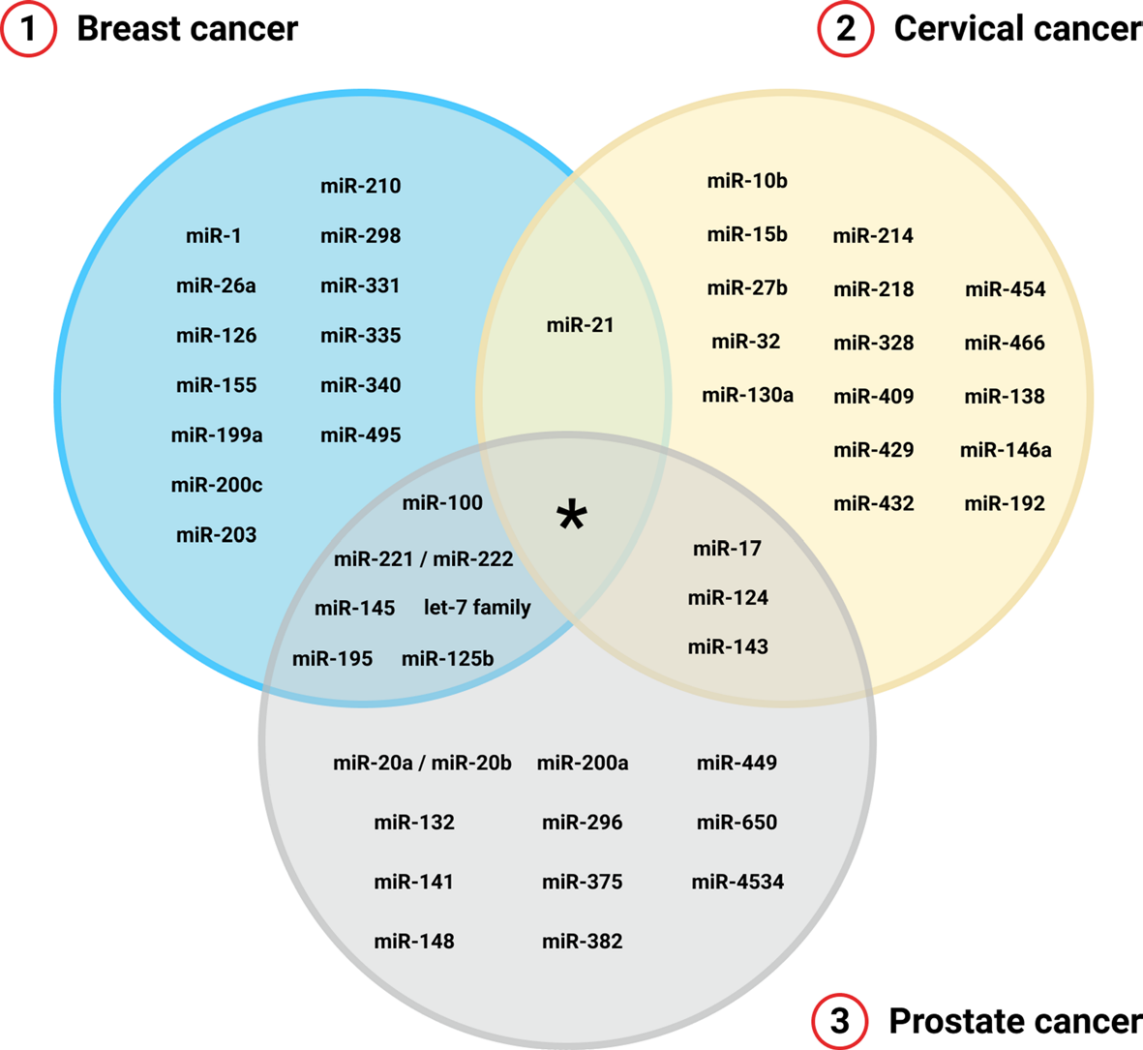
Representation in a Venn diagram of the sharing or differentially expressed miRNA profile in only one type, according to the proposed panels for (1) breast; (2) cervical; or (3) prostate cancer. The star represents the absence of over position on miRNA profile mutually altered in all three cancer types according to the literature reviewed. This possibility, however, was not excluded if we consider the expression of other miRNAs not presented in the tables

In this sense, the expression of miR-17, miR-20a/miR-20b, miR-148, miR-650, and miR-4534 are associated with induction of cell differentiation and tumor angiogenesis [134–137]. On the other hand, the expression of let-7 family, miR-100, miR-124, miR-125b, miR-132, miR-141, miR-143, miR-145, miR-195, miR-200a, miR-221, miR-296, miR-375, miR-382, and miR-449 are associated with the inhibition of cell proliferation and metastasis formation [94, 138–165]. Table 3 summarizes these findings associated in the development of prostate cancer. Figure 2 illustrates in a Venn diagram the sharing or differentially expressed miRNA profile in only one type of cancer, according to the data present in the tables. The star represents that, considering only these panels, there is no over position on miRNA expression mutually altered in all three cancer types according to the literature reviewed. However, this possibility was not excluded if we considered the expression of other miRNAs not presented in the tables.

**Table 3:** Preliminary panel for miRNA quantification related to prostate cancer development

miRNA	Cellular function	References
let-7 family	Inhibition of cell proliferation, migration, and metastasis	[138–140]
miR-17	Promotion of cellular proliferation and tumor angiogenesis	[134]
miR-20a/miR-20b	Regulation of cell proliferation, differentiation, and apoptosis	[134]
miR-100	Inhibition of tumorigenesis, cell proliferation, and signaling	[141]
miR-124	Inhibition of cellular proliferation and migration	[142, 143]
miR-125b	Inhibition of cellular proliferation and induction of metastasis	[144, 145]
miR-132	Inhibition of cellular proliferation and signaling	[146]
miR-141	Inhibition of cellular proliferation and migration	[94, 147]
miR-143	Regulation of apoptotic mechanisms	[148, 149]
miR-145	Inhibition of cellular proliferation, migration and tumor angiogenesis	[150, 151]
miR-148	Regulation of angiogenesis and apoptotic mechanisms	[135]
miR-195	Inhibition of cellular proliferation, cell cycle progression, and metastasis	[152, 153]
miR-200a	Inhibition of cellular proliferation and signaling	[154, 155]
miR-221	Inhibition of cell proliferation and invasion	[156–158]
miR-296	Inhibition of cell proliferation and invasion; induction of apoptotic mechanisms	[154, 159, 160]
miR-375	Inhibition of cell proliferation and invasion	[161, 162]
miR-382	Inhibition of cell proliferation, migration, and metastasis	[163]
miR-449	Control of cell proliferation and differentiation	[164, 165]
miR-650	Regulation of pro-inflammatory signals and induction of cell proliferation	[136]
miR-4534	Induction of cell migration and metastasis	[137]

### Current strategies adopted to quantify miRNAs and major challenges to the development of new diagnostic tools in the IoT era

Besides millions of deaths worldwide, the cancer diagnosis also has a huge economic impact on individuals and health systems. According to recent data from the National Cancer Institute—NIH, the annual cost of treatment for breast, prostate, and cervical cancer is estimated at 35.6 billion dollars, mainly directed to hospitalizations, therapeutic agents, and palliative care. Additionally, the indirect impact associated with the lost productivity associated with cancer diagnosis represents an extra amount of 17.2 billion dollars per year [166–168].

Among the main challenges to the dissemination of new tools for the accurate early cancer diagnosis are the use of expensive methods/equipment and the need for specialized professionals, which makes unfeasible their application in low- and middle-income countries and remote locations. The advances observed in cancer therapy changed the outcome and improved the quality of life during the treatment, evolving from a death sentence to a curable disease in several cases in only a few decades. However, despite the efforts, the diagnostic tests do not go along with these advances or are expensive to be applied as screening methods in primary health care [92, 93].

Usually, the cancer diagnostic methods are based on high-resolution imaging, detection of metabolites in tissue samples/biological fluids, or morphological/phenotypic cellular analysis. Nevertheless, even when all those requirements are available, it does not guarantee the establishment of a precise early cancer diagnosis due to the stage of disease development, lack of sensibility or misinterpretation of requested exams, and false-positive or false-negative results [10].

In recent decades, the initial techniques to detect miRNAs were optimized and progressively replaced by more accurate and less laborious methods [94]. Currently, the main techniques used for miRNA quantification are based on microarray platforms, qPCR, next-generation sequencing, Northern blotting, or isothermal amplification. Among these methods, those based on microarray platforms and qPCR are widely used in basic researches and manufactured by different companies. In general, both strategies adopt a labeled-probe strategy to quantify their targets using optical sensors and report the results. However, the elevated operational cost of analysis and the multiple-step processing samples are issues that need to be solved to allow the scalability of these methods using clinical samples.

On the other hand, the application of next-generation sequencing, Northern blot, and isothermal amplification for miRNA quantification requires multiple-step protocols and they are more expensive than microarray platforms and qPCR, especially when we analyze multiple targets simultaneously. Table 4 summarizes the main features of each method and current limitations for their application as a screening method for early cancer detection. Considering these features, recent papers that quantify miRNAs in clinical samples aiming to detect potential biomarkers are mostly based on microarrays platforms and qPCRs strategies, regarding their better processing scalability, analysis of results, and overall costs in multiple targets assays [80, 92, 93, 101].

**Table 4:** Main methods used for miRNA quantification and current limitations for their application as a screening method for cancer detection

Method	Technique	Advantages of technology	Limitations	Sensibility	Reference
Microarray platforms	Direct hybridization with probes present in the platform	Potential scalability; quantitative results; few steps until final results	Needed of specialized professionals and equipment; requires the previous setup of miRNA panel; elevated costs of customized panel	fmol	[178, 179]
qPCR	miRNA amplification and quantification; results were detected using optical sensors	Widespread technique; quantitative results; few steps until final results	Needed of specialized professionals and equipment; time until final results; requires the previous setup of miRNA panel	fmol	[162]
Next-generation sequencing	Direct sequencing of miRNAs present in the sample	Quantification of all miRNAs in the sample; high accuracy	Elevated operational cost of analysis; needed of specialized professionals and equipment; time until final results	fmol	[180, 181]
Northern blot	Hybridization with probes and detected according to their sequences and molecular weight	Widespread technique	Semi-quantitative method; laborious and multiple steps protocol	pmol	[182, 183]
Isothermal amplification	miRNA amplification and quantification; results were detected using optical sensor	Low-cost technique; potential scalability	Nonspecific background amplification; multiple steps protocol; Scalability using clinical samples	fmol	[184–186]

**Fig. 3:**
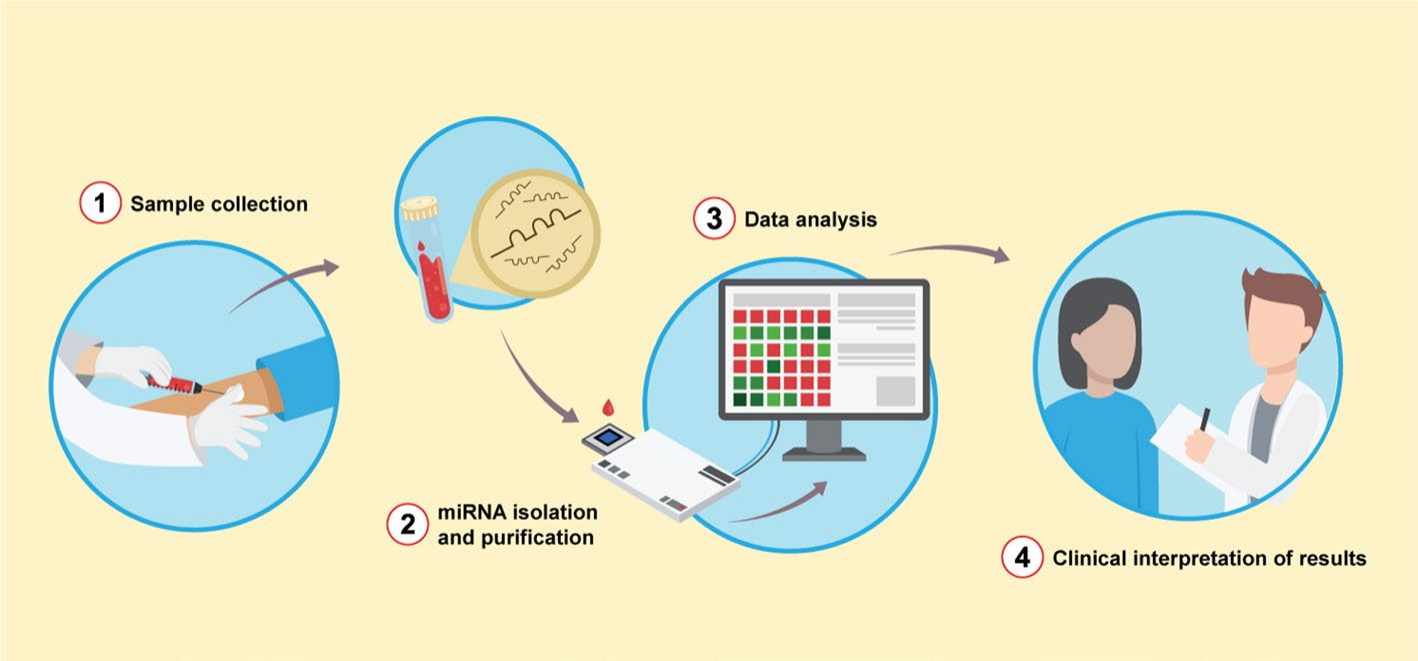
Development of a low-cost platform to quantify miRNAs using minimally invasive samples and their application as a diagnostic tool for cancer screening. Among the desirable features for the application of a new diagnostic tool, this strategy may reduce the technical barriers to early cancer diagnosis and contribute to the application of miRNAs quantification as a powerful screening method. (1) Use of minimally invasive samples. (2) Few steps of sample processing until the complete miRNA isolation. (3) miRNA quantification and data analysis. (4) Clinical interpretation of results and effective communication of them between the health professional and the patient

In this sense, one of the major challenges on miRNA field is to apply the knowledge from different areas (as medicine and biomedical engineering) to the development of a trusted, cheap and portable platform/devices, which uses minimally invasive samples (i.e., serum/plasma, saliva or urine) to contribute for early cancer detection. However, beyond the inherent biological aspects, the development of these new tools should also consider their application as a smart device and be able to connect with other medical devices and to the Internet of Things (IoT). An efficient low-cost platform using minimally invasive samples, as represented in Fig. 3, which with few steps since the minimally invasive sample collection (step 1), sampling processing (step 2), miRNA quantification (step 3) through the final communication of results (step 4) can reduce these technical barriers and contribute to the democratization of access to cancer screening tests, even in remote populations. The elaboration of a complex panel based on updated results from several platforms and the device connection with specialized centers could be useful to test isolated populations and optimize the analysis of results, contributing to developing personalized medicine strategies and translating the knowledge from bench to save lives.

Taken together, these advances may represent a new era in cancer diagnosis procedures. As observed in several industry segments, the revolution 4.0 in health practices and devices can subvert the rationale of investments (from hospitalization to primary care assistance), contributing to early diagnosis and optimizing the resources available [95, 169, 170].

## Conclusion

Considering the progressive number of new cancer cases every year worldwide, the development of new tools to improve the early detection methods is critical to improving the sensitivity and specificity in screening tests to allow a better prognosis for these patients. Besides the deaths associated with late diagnosis, cancer therapy has a huge economic impact associated with therapies, prolonged hospitalization periods, and the occurrence of metastasis. Currently, several papers associated changes in the miRNA expression to the development of tissue-specific modifications and aberrant cellular proliferation. Although there is a lack in the consensus on the miRNA expression between tissue and biological fluid samples, the development of new studies and the establishment of an adequate biomarkers panel may be useful to detect several cancer types using minimally invasive samples. The current methods used to quantify miRNAs in clinical samples frequently are expensive, based on multiple steps protocols and demand a high-specialized professionals, making their large-scale application unfeasible.

In this sense, the application of miRNAs as biomarkers of early cancer development may contribute to the development of precision medicine and improve life expectancy and quality of life from affected patients. However, there is still a challenge to make the miRNA quantification an economically feasible approach to be adopted widely in primary health care. The development of new technologies and/or optimization of current strategies adopted to quantify these molecules applied into a new portable device able to detect these molecules using blood or other biological fluids could represent an important strategy to democratize, with agility and excellence, the cancer screening strategy and take the next step forward to the development of personalized medicine.

## Data Availability

All data generated or analyzed during this study are included in this published article.

## Abbreviations

Bcl-2: B-cell lymphoma 2 protein
CD8+ T cells: Cytotoxic lymphocyte
CNS: Central nervous system
dsRNA: Double-strand RNA
ER-α/β: Estrogen receptor-alpha/beta
FFPE: Formalin-fixed paraffin-embedded
IoT: Internet of things
mRNAs: Messenger RNAs
miRNAs: MicroRNAs
miR-RISC: MicroRNA-induced silencing complex
NIH: National Institutes of Health
NK cells: Natural killer cells
nt: Nucleotides
onco-miRs: Oncogenic miRNAs
p53: Cellular protein 53
PSA: Prostate-specific antigen
PTEN: Phosphatase and tensin homolog protein
qPCR: Quantitative polymerase chain reaction
RNA: Ribonucleic acid
RECK: Reversion-inducing cysteine-rich protein with Kazal motifs
Treg cells: Regulatory T cells
